# Beyond Body Mass Index: Are Weight-loss Programs the Best Way to Improve the Health of African American Women?

**DOI:** 10.5888/pcd14.160573

**Published:** 2017-06-15

**Authors:** Leilani Dodgen, Emily Spence-Almaguer

**Affiliations:** 1 University of North Texas Health Science Center, Fort Worth, Texas

## Abstract

African American women have higher prevalence (82%) of overweight (body mass index [BMI] 25–29) and obesity (BMI ≥30) than white women (63.2%) or Hispanic women (77.2%), and weight-loss programs yield minimal results in this population. We examine the concept of BMI as a measure of health for African American women and suggests a more holistic, multifaceted approach to preventing chronic disease.

## Background

Rates of chronic disease, disability, and premature death among African American women are disproportionately high. Compared with non-Hispanic white women, African American women are 1.9 times as likely to develop diabetes, 2.3 times as likely to develop end-stage kidney disease, and 2.4 times as likely to die from complications of diabetes (1). Hypertension, high cholesterol, obesity, tobacco use, excessive alcohol consumption, and poor diet are known risk factors for chronic disease, and African American women experience these diseases at higher rates than white women experience them (1,2). Because African American women aged 20 years or older have higher prevalence of overweight and obesity (82%) than white women (63.2%) or Hispanic women (77.2%), weight loss is regarded as the way to improve their health (3).

Body mass index (BMI) (kg/m^2^) is widely used in medical practices, health promotion programs, and research as a measure of health because it is easy to calculate and involves no costly procedures (4). The prevalent use of BMI combined with the US obsession with weight made BMI a primary choice to serve as an indicator of chronic disease risk and a measure of health. However, using BMI reduces the complexities of health to one primary indicator and its accompanying solution, weight loss. Multiple weight-loss programs for African American women over the past 20 years show minimal results. Compared with white women, African American women in the same research programs lose less weight and maintain weight loss for shorter time (5).

## Body Mass Index Is Difficult to Change

In weight-loss programs, African American women are unlikely to transition from obese (BMI ≥30) or overweight (BMI 25–29) to a normal body weight (BMI <25). The average African American woman weighs 85 kg, has a BMI of 32.2, and would need to lose more than 8 kg to reach the top of the overweight threshold (BMI 29). Most participants in weight-loss programs do not lose this much weight (6–8). Research suggests that sustained weight loss of 5% to10% of body mass can reduce risk factors for cardiovascular disease and prevent progression toward diabetes (9); however, on average, African American women participating in a weight-loss program do not lose this much weight. A review of weight-loss programs (6), showed the average weight loss of African American women was 1.9 kg to 4.7 kg compared with 4.7 kg to 7.5 kg for white women. To lose 5% to 10% of body weight, an 84 kg woman needs to lose 4 kg to 8 kg. 

Maintaining weight loss is also difficult. In a review of weight-loss programs with a maintenance phase, at 18-month follow up African American women regained from 0% to 33% of weight previously lost (10). If African American women lose on average 4.5 kg (the highest weight loss reported in most programs) excluding possible weight regain, only people who are borderline between categories (eg, between overweight and normal) would move to a lower BMI category. African American women are held to the BMI standard as a measure of health despite the inability of interventions to change BMI.

To help African American women lose weight, interventions have been tailored to an African American audience by using culturally appropriate artwork and language; African American media outlets; formative assessments (eg, focus groups, interviews); and meaningful themes such as traditional foods, family, and spirituality (7). However, results from such interventions were small, and average weight loss ranged from 2.0 to 4.5 kg (7). Interventions for African American women achieve minimal weight loss and for short periods (usually 6 months). Also, because researchers most commonly report only mean weight loss, only a few participants may have had any weight loss (6,7,10). To guide future research, more information is needed regarding the percentage of participants who meet weight-loss goals rather than mean weight loss for an overall program.

## Body Mass Index Is a Poor Proxy for Health

BMI is often the standard by which health care providers assess women’s health, and it is the basis for planning health improvements (4). However, BMI is a poor proxy for general health and health behaviors because it fails to account for differences in body composition, fitness levels, and nutritional differences that are predictive of health and longevity (11). People can be obese but in good health because of diet and physical activity. Furthermore, a healthy BMI may seem out of reach to African American women who have difficulty losing weight or maintaining weight loss (12).

Although achieving a normal BMI is often unsuccessful, this is not due only to ineffective interventions. BMI itself as a measure of health is of limited value. For example, studies show that BMI misclassifies people with large muscle mass (13). Alternatives to BMI include measuring total body fat and percentage of body fat, even though these are more difficult to measure (13). Nevertheless, such alternatives need to be considered when evaluating the health of African American women, and weight-loss goals may need to be adjusted for muscle mass.

BMI is often used as a measure of behavioral or lifestyle risk. For example, deaths attributable to health behaviors were estimated by combining mortality data and prevalence data from the National Health and Nutrition Examination Surveys (NHANES) (2). In this seminal 2004 study, the second highest cause of death was “poor diet and physical inactivity” (2). However, this assessment was made by using BMI as a proxy for physical activity and poor nutrition (2). These results are misleading because BMI is not a logical equivalent of nutrition quality or physical activity. Because the NHANES dataset (1999–2000) included measures of diet and physical activity, it is unclear why BMI was used as a proxy for these constructs, particularly because later studies demonstrate that physical activity independently influences mortality (11,14). Furthermore, an analysis of more recent NHANES data (2005–2012) indicated that using the BMI to predict cardiometabolic risk misclassifies nearly 75 million US adults, of whom more than 54 million are overweight or obese but have healthy cardiometabolic indicators (15).

Alternatives to BMI as a measure of health and disease risk are available. Research increasingly points to measures of fitness over fatness as an indicator of health and well-being and shows that people who are overweight or obese and who have good cardiorespiratory fitness have similar mortality rates to people with normal BMI and good cardiorespiratory fitness levels (11). In fact, mortality was shown to be dependent on cardiorespiratory fitness and not BMI (11). Thus a high BMI does not automatically mean a greater risk of death. This is good news for people who are unsuccessful at losing weight or maintaining weight loss. A moderate level of physical activity can improve health regardless of whether a person loses weight (11). In measuring health and risk, not all measures account for differences in muscle mass or fitness between individuals.

## Body Mass Index May Not Affect Health Behavior of African American Women

Health care providers should consider motivations for achieving and maintaining health, because motivation drives behavior (11). Among African American women, a common perception is that big is beautiful. Studies document this social norm, showing that often, although not always, African American women prefer a full and curvier shape than other US women. Compared with white women, African American women view a large body as preferable and as more attractive than a thin body, and cultural and social norms in the African American community support this view (12). African American women report that large body size can be attractive and healthy, and they often do not perceive themselves as overweight. Such beliefs and social norms discourage weight loss because size is perceived as an asset (12).

Although body size may not be a motivator for weight loss, African American women frequently cite health as the most important reason to lose weight (12). In focus groups, they expressed a desire to prevent many of the diseases they saw in their families (12). One study reported that African American women conceptualize health as multifaceted, as including mind, body, soul, and environment, and perceived health as both in and out of their control (eg, because of environmental factors) (16). If health is the reason African American women want to lose weight, then researchers need to understand concepts of health from the perspectives of African American women. Only by doing so can researchers develop programs and partnerships that help African American women achieve their goal of preventing chronic disease.

## Body Mass Index Minimizes the Complexity of Health

By focusing primarily on weight loss and BMI, other avenues toward better health are minimized. Health is complex and multifaceted; individual behaviors are estimated to contribute approximately 40% to the risk for premature death, and environment and social factors make up another 20% (17). Obesity prevention needs to expand in interventions to address factors beyond the individual to the environmental and social contexts of participants’ lives to influence the social determinants of health (6).

Many factors beyond eating habits and inactivity contribute to obesity among African American women. At the community level, poverty and neighborhood environment are associated with increases in weight and rates of obesity (6,18). Data from the Office of Minority Health, US Department of Health and Human Services, (1) show that the percentage of African Americans who live below the federal poverty level (28.1%) is more than twice the percentage of whites who do so (11%). Living in a racially segregated black neighborhood also increases risk of obesity for black women (18). Although segregation is decreasing, even middle class African Americans are still more likely than middle class whites to live in poor-quality neighborhoods (18). Additionally, both food preferences and norms related to body size are shaped by social norms in the environment (18).

At the social and interpersonal levels, racism and perceived discrimination also contribute to low rates of weight loss and high obesity rates among black women. The Black Women’s Health Study reported mean weight and waist circumference increased in association with experienced racism at all levels of neighborhood segregation (18). This means that, regardless of which part of town a woman lives in, experiencing racism is positively associated with obesity. This finding may help to explain why obesity is more prevalent among African American women than among women in other groups.

Identity also plays a role in obesity. The strong black woman or superwoman identity grew out of the African American experience of slavery and continues even today, characterized by “obligation to manifest strength, obligation to suppress emotions, resistance to being vulnerable or dependent, determination to succeed despite limited resources, and obligation to help others” (19). The burden of this identity often manifests in stress leading to coping mechanisms such as emotional eating that may end in weight gain. One study (19) found that African American women who more often hid or denied their struggles to portray an image of strength, were also reported to have higher levels of obesity (BMI ≥30). Stress and strain associated from maintaining a strong identity contributes to many adverse health indicators.

Conversely, identity may also be a motivator for change. One study showed that a strong racial identity among African American women was associated with increased motivation to exercise but was not associated with motivation to lose weight. Recognizing the crucial influence of physical activity on health, programs that engage African American women in pursuing their own self-selected health goals may be of more value than those with prescribed weight-loss expectations (20). The ways that identity helps or hinders health behaviors need to be further explored. New solutions that focus on the whole person at all levels, from family unit to environment, are needed to transform not just the health of African American women, but also to transform their families, neighborhoods, and ultimately their communities.

## Community-Based Participatory Research Provides a Broader, More Holistic Approach to Health

One approach that focuses on the whole person at all levels is community-based participatory research (CBPR) (Box). CBPR is “a collaborative approach to research that equitably involves all partners in the research process and recognizes the unique strengths that each brings. CBPR begins with a research topic of importance to the community and proceeds to combine knowledge with action to achieve social change, to improve health outcomes, and to eliminate health disparities” (21).

**Box. Principles of Community-Based Participatory Research**

1. Recognizes community as a unit of identity.
2. Builds on strengths and resources within the community.
3. Facilitates collaborative partnerships in all phases of the research.
4. Integrates knowledge and action for mutual benefit of all partners.
5. Promotes a co-learning and empowering process that attends to social inequalities.
6. Involves a cyclical and iterative process.
7. Addresses health from both positive and ecological perspectives.
8. Disseminates findings and knowledge gained to all partners.

Source: Israel et al (28)

Although many interventions have used community-engaged or participatory approaches in obesity interventions, their partnerships did not have the characteristics of mature CBPR, namely a community-identified issue of interest and involvement in *all* phases of the research from development to dissemination, a commitment to co-learning, a focus on community capacity-building, and a focus on social change over a long period (22,23). Many programs make limited use of the CBPR approach, only using advisory boards or focus groups during the initial phases of program development, when assistance with recruitment and implementation from a community lay health promoter or health advisor would be most useful and informative, then lessening engagement with partners as the study progresses into later phases (7). The outcomes of these studies are similar to those of other tailored approaches to obesity prevention that show the same lower ranges of weight loss (6,7, 24).

CBPR in its intended form, as an approach in all phases of the research, is a framework for programs that is especially suitable for the participants and transformative for the community (25). The CPBR approach aligns with what researchers of African American women’s health call an “embodied approach to investigating health disparities [that] moves beyond rational explanations and incorporates contextual factors emphasizing how an individual’s subjective experience influences health behaviors” (26). The subjective experience of African American women as partners in research is needed to promote their strengths and to address the multiple dimensions that contribute to poor health and chronic disease. CBPR enables researchers and community partners to focus on context, and the assets and resiliencies of African American women can be captured to produce programs that are meaningful to them instead of programs that focus on weight loss alone. For example, in one mature CBPR study with African American women, the collaborative team ultimately shifted its focus from using weight loss alone as a metric to what they labeled a common sense approach more focused on physical activity and nutritional goals perceived by participants as relevant and valuable (27).

A broader, more holistic approach to health that includes the values of African American women may lead to greater reductions in chronic disease and a shift beyond BMI to create solutions that embody the complexity of staying or becoming healthy and that aim for health equity for African American women and their communities.
